# Carbon Assimilation Strategies in Ultrabasic Groundwater: Clues from the Integrated Study of a Serpentinization-Influenced Aquifer

**DOI:** 10.1128/mSystems.00607-19

**Published:** 2020-03-10

**Authors:** Lauren M. Seyler, William J. Brazelton, Craig McLean, Lindsay I. Putman, Alex Hyer, Michael D. Y. Kubo, Tori Hoehler, Dawn Cardace, Matthew O. Schrenk

**Affiliations:** a Marine Chemistry and Geochemistry, Woods Hole Oceanographic Institution, Woods Hole, Massachusetts, USA; b Blue Marble Space Institute of Science, Seattle, Washington, USA; c Department of Biology, University of Utah, Salt Lake City, Utah, USA; d Department of Earth, Atmospheric and Planetary Sciences, Massachusetts Institute of Technology, Cambridge, Massachusetts, USA; e Department of Earth and Environmental Sciences, Michigan State University, East Lansing, Michigan, USA; f Space Sciences Division, NASA Ames Research Center, Mountain View, California, USA; g Department of Geosciences, University of Rhode Island, Kingston, Rhode Island, USA; h Department of Microbiology and Molecular Genetics, Michigan State University, East Lansing, Michigan, USA; Lawrence Berkeley National Laboratory

**Keywords:** carbon assimilation, carbon fixation, formaldehyde, formate, methane, serpentinization

## Abstract

Serpentinization is a low-temperature metamorphic process by which ultramafic rock chemically reacts with water. Such reactions provide energy and materials that may be harnessed by chemosynthetic microbial communities at hydrothermal springs and in the subsurface. However, the biogeochemistry mediated by microbial populations that inhabit these environments is understudied and complicated by overlapping biotic and abiotic processes. We applied metagenomics, metatranscriptomics, and untargeted metabolomics techniques to environmental samples taken from the Coast Range Ophiolite Microbial Observatory (CROMO), a subsurface observatory consisting of 12 wells drilled into the ultramafic and serpentinite mélange of the Coast Range Ophiolite in California. Using a combination of DNA and RNA sequence data and mass spectrometry data, we found evidence for several carbon fixation and assimilation strategies, including the Calvin-Benson-Bassham cycle, the reverse tricarboxylic acid cycle, the reductive acetyl coenzyme A (acetyl-CoA) pathway, and methylotrophy, in the microbial communities inhabiting the serpentinite-hosted aquifer. Our data also suggest that the microbial inhabitants of CROMO use products of the serpentinization process, including methane and formate, as carbon sources in a hyperalkaline environment where dissolved inorganic carbon is unavailable.

**IMPORTANCE** This study describes the potential metabolic pathways by which microbial communities in a serpentinite-influenced aquifer may produce biomass from the products of serpentinization. Serpentinization is a widespread geochemical process, taking place over large regions of the seafloor and at continental margins, where ancient seafloor has accreted onto the continents. Because of the difficulty in delineating abiotic and biotic processes in these environments, major questions remain related to microbial contributions to the carbon cycle and physiological adaptation to serpentinite habitats. This research explores multiple mechanisms of carbon fixation and assimilation in serpentinite-hosted microbial communities.

## Abstract (precis)

This study describes the potential metabolic pathways by which microbial communities in a serpentinite-influenced aquifer may produce biomass from the products of serpentinization. Serpentinization is a widespread geochemical process, taking place over large regions of the seafloor and at continental margins, where ancient seafloor has accreted onto the continents. Because of the difficulty in delineating abiotic and biotic processes in these environments, major questions remain related to microbial contributions to the carbon cycle and physiological adaptation to serpentinite habitats. This research explores multiple mechanisms of carbon fixation and assimilation in serpentinite-hosted microbial communities.

## INTRODUCTION

Serpentinization is the process by which ultramafic rock in the lower crust and upper mantle of the Earth is hydrated, leading to the oxidation of ferrous iron in the minerals olivine and pyroxene and the release of hydrogen (H_2_) and hydroxyl ions (OH^−^). The reducing power supplied by serpentinization, when mixed with oxidants from surface and subsurface fluids, creates chemical disequilibria that microbial populations can harness (1–3). However, due to high pH and the release of cations during rock weathering, much of the dissolved inorganic carbon (DIC) in serpentinite systems is precipitated as calcite and aragonite minerals (4). Consequently, small carbon-bearing compounds, including methane (CH_4_), carbon monoxide (CO), and formate, may take on important roles in sustaining microbial ecosystems in serpentinites (5). The high pH and limited availability of both DIC and electron acceptors represent fundamental challenges for microbial communities hosted in serpentinization-influenced environments (6).

**FIG 1:**
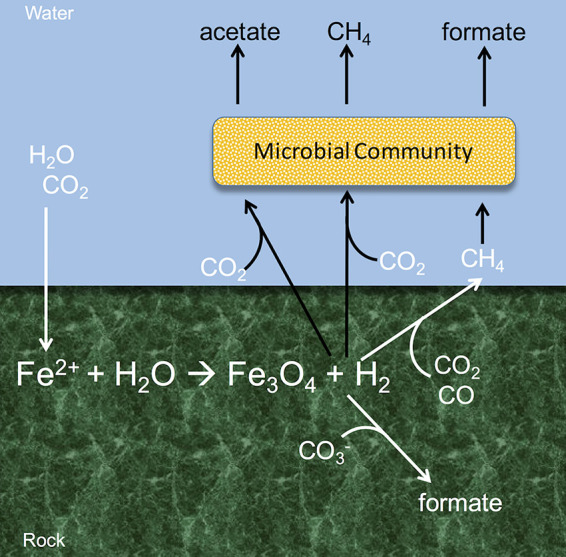
Diagram depicting abiotic (white) and biotic (black) sources of carbon compounds in serpentinizing environments. Adapted from the work of Preiner et al. (83).

Overlapping biogenic and abiogenic processes in serpentinizing rocks make it difficult to delineate carbon cycling pathways used in these environments (Fig. 1). Under highly reducing conditions, in the presence of specific mineral catalysts, hydrogen reacts with carbon dioxide (CO_2_) or carbon monoxide to form methane and small-chain hydrocarbons via Fischer-Tropsch-type reactions (7–10). These compounds are also formed by biological activity, or diagenesis of organic matter (11). For example, high concentrations of dissolved organic material have been detected in close association with serpentine-hosted hydrogarnets recovered from the Mid-Atlantic Ridge. This material may have been produced by past microbiological activity (12) or by purely abiotic mechanisms (13). Acetate in endmember fluids at the Lost City Hydrothermal Field is likely produced by biological activity (14), but other hydrocarbons in the rock-hosted fluids do not have a clear biotic or abiotic source (15), though formate and C_2_^+^ alkanes appear to be produced abiotically (9, 14). Biotic versus abiotic sources of methane in serpentinizing systems are likewise difficult to resolve and vary from site to site (16–19).

In situ microbial communities may utilize the products of serpentinization—including methane, carbon monoxide, formate, and acetate—as sources of carbon (14, 20–25). Microbial communities in serpentinite springs in the Voltri Massif, Italy, appear to perform both aerobic methanotrophy and methanogenesis using CO_2_ liberated from acetate (26). Methanogens in The Cedars, CA, are capable of producing methane from both inorganic carbon and a variety of organic carbon substrates. Communities that reside deeper in the springs at The Cedars also possess a reductive acetyl coenzyme A (acetyl-CoA) pathway for autotrophic acetogenesis (27). Fermentation of organic matter may also lead to acetate production (19). Carbon monoxide may be used as a substrate by microbial communities in the Tablelands, Canada, but it appears to be primarily used as a source of energy rather than carbon (28). The carbonate chimneys of the Lost City Hydrothermal Field are dominated by a single species of archaea that may be capable of both production and consumption of methane in tandem (21). Genes encoding NAD(+)-dependent formate dehydrogenase in metagenomes recovered from the Samail Ophiolite, Oman, aquifer suggest that formate is an important source of carbon (29). In short, sources and sinks of carbon in these environments remain enigmatic to researchers, but pathways and strategies utilizing alternate sources of carbon supplied by serpentinization appear to be prevalent.

In this study, we examined the microbial metabolic potential and biogeochemistry of a serpentinization-influenced aquifer in the Coast Range Ophiolite Microbial Observatory (CROMO), CA. Wells have been drilled at various depths and locations at CROMO such that microbial processes can be explored across a wide range of physical and chemical conditions. Carbon assimilation pathways in the microbial communities at CROMO were investigated using a combination of metagenomic, metatranscriptomic, and metabolomic approaches. Metagenomic data sets from 9 of the 12 CROMO wells, and metatranscriptomic data sets from 4 wells, obtained over multiple sampling trips, were queried for complete pathways and individual genes associated with carbon fixation and methane cycling. We then applied environmental metabolomics to directly assess the products of ecosystem activity in intra- and extracellular extracts from the two most alkaline wells (CSW1.1 and QV1.1). This study linked the genetic potential of serpentinite-hosted communities to the observed biogeochemistry of the groundwater in CROMO.

## RESULTS

### Presence and expression of carbon fixation and assimilation pathways.

**FIG 2:**
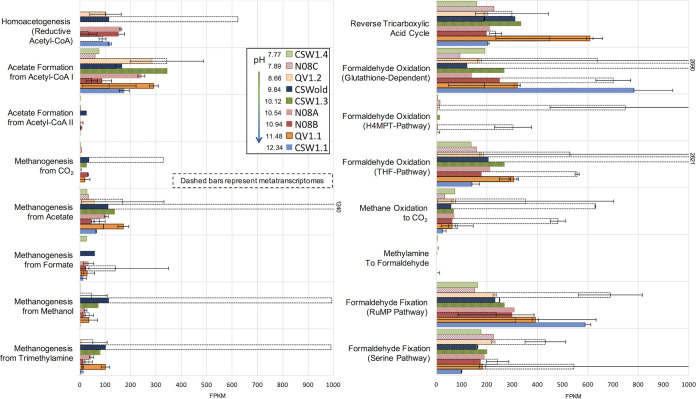
Metagenome coverage of Functional Ontology Assignments for Metagenomes (FOAM) pathways assigned using HUMAnN2. If a well was sampled for metagenomics more than once, the average percent coverage and standard error of the mean for that well are provided. Metatranscriptome coverage for four wells sampled for RNA is indicated using bars with dotted outlines.

**FIG 3:**
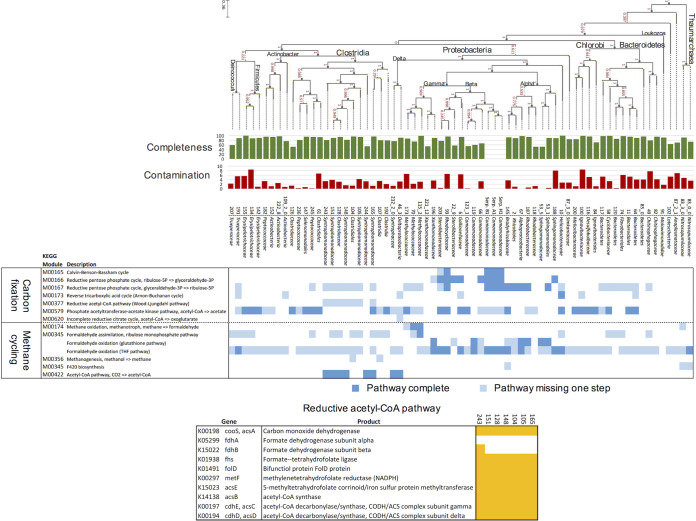
Selection of carbon cycling-associated KEGG modules detected in MAG bins, and *Serpentinomonas* isolate genomes (69), using BLASTKoala. Dark blue boxes indicate that the complete module is present; light blue boxes indicate that one step of the pathway is missing. The phylogenetic tree comparing the MAGs to the three *Serpentimonas* isolates was generated using SpeciesTree in KBase. Completeness and contamination scores for each MAG are also provided. (Bottom) Bins containing the reductive acetyl-CoA pathway; gene presence in the genome is indicated in yellow.

The metagenomic data collected from 9 of the 12 CROMO wells contain evidence for a range of carbon fixation and assimilation pathways (Fig. 2). Functional profiling of the metagenomes using HUMAnN2 identified multiple C_1_ compound utilization and assimilation pathways, including the reductive acetyl-CoA (Wood-Ljungdahl) pathway, the reverse tricarboxylic acid (rTCA) cycle, formaldehyde oxidation to formate, and formaldehyde fixation to biomass. Whenever these complete pathways were identified in the metagenomic data, they were also detected in the corresponding metatranscriptomic data (Fig. 2). A complete Calvin-Benson-Bassham (CBB) cycle was also detected in all nine metagenomes and all four metatranscriptomes, at similar coverages across wells (see Fig. S1 in the supplemental material). Carbon fixation pathways found exclusively in archaea (i.e., hydroxypropionate-hydroxybutylate cycle and 3-hydroxypropionate bicycle) were not detected. Metagenome-assembled genome (MAG) bins contained the reductive pentose phosphate/Calvin-Benson-Bassham cycle, reductive TCA cycle, and reductive acetyl-CoA pathways (Fig. 3).

10.1128/mSystems.00607-19.1 FIG S1 Fragments per kilobase of sequences per million mapped reads (FPKM) of genes in the Calvin-Benson-Bassham cycle. Metagenomes are represented in black; metatranscriptomes are represented in gray. Download FIG S1, PDF file, 0.2 MB. Copyright © 2020 Seyler et al. 2020 Seyler et al. This content is distributed under the terms of the Creative Commons Attribution 4.0 International license.

**FIG 4:**
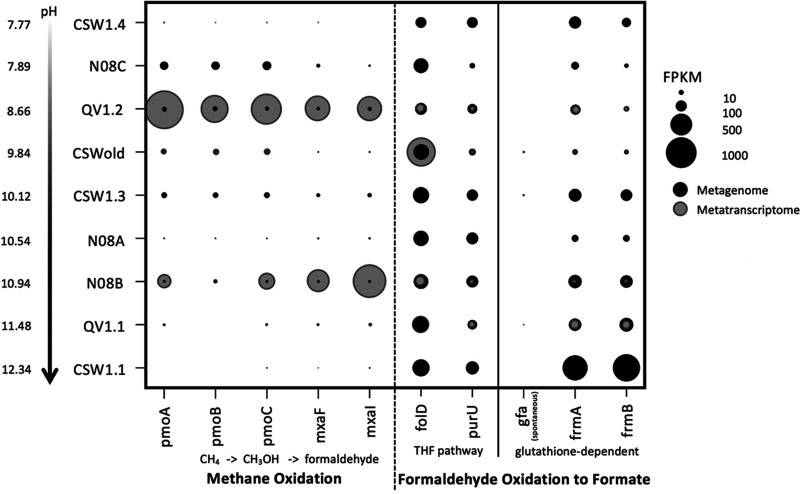
Fragments per kilobase of sequences per million mapped reads (FPKM) of genes in the homoacetogenic reductive acetyl-CoA pathway. Metagenomes are represented in black; metatranscriptomes are represented in gray. The genes for the two subunits of formate dehydrogenase (*fdhA* and *fdhB*) are missing or comparatively low in most wells. This enzyme is responsible for converting CO_2_ to formate.

Several pathways associated with methanotrophy and methylotrophy were detected in the metagenomes and metatranscriptomes. The complete pathway for aerobic methane oxidation to formaldehyde was present in most of the metagenomes (Fig. 4) and transcribed in wells QV1.2 and N08B, two of the shallower, more oxic wells in the observatory. Formaldehyde uptake and assimilation pathways were detected in all of the sequenced wells (Fig. 2). Genes for the complete glutathione-dependent and tetrahydrofolate (THF) pathways for formaldehyde oxidation to formate were present in all of the metagenomes, expressed in all four metatranscriptomes (Fig. 2 and 4), and present in multiple MAGs (Fig. 3). The *gfa* gene, which codes for an enzyme that catalyzes a thermodynamically spontaneous step in the glutathione-dependent pathway (30), is found in only some formaldehyde-oxidizing bacteria and was absent from most of the metagenomes. Genes for formaldehyde fixation via the tetrahydromethanopterin (H4MPT) pathway had low coverage across the metagenomes but were expressed in the metatranscriptomes of QV1.2 and N08B. The capability for formaldehyde fixation to biomass by the ribulose monophosphate (RuMP) and serine pathways was likewise suggested in all nine metagenomes and all four metatranscriptomes (Fig. 2), and the RuMP pathway was partially complete in 18 MAGs and complete in 1 MAG (Fig. 3).

**FIG 5:**
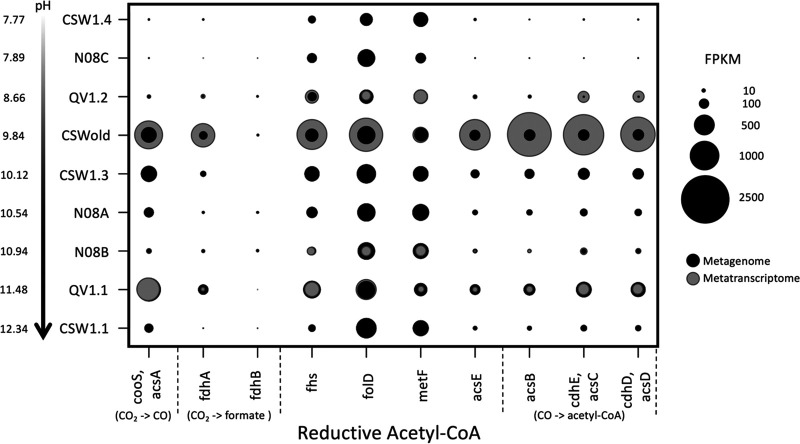
Fragments per kilobase of sequences per million mapped reads of genes in the methane oxidation, tetrahydrofolate (THF), and glutathione-dependent formaldehyde oxidation pathways. Metagenomes are represented in black; metatranscriptomes are represented in gray. The gene for *S*-(hydroxymethyl)glutathione synthase (*gfa*) is present at low abundance or missing in all wells; this enzyme catalyzes a spontaneous reaction in the formaldehyde oxidation pathway.

Pathway identification using the Kyoto Encyclopedia of Genes and Genomes (KEGG) database and Functional Ontology Assignments for Metagenomes (FOAM) pathways suggested that homoacetogenesis via the reductive acetyl-CoA pathway (also known as the Wood-Ljungdahl pathway) was represented in the metagenomes of only half of the wells sequenced (N08A, N08B, QV1.2, CSW1.1, and CSWold) (Fig. 2). However, a gene-by-gene search indicated that the pathway was also present, and nearly complete, in the QV1.1 and CSW1.3 wells (Fig. 5 and Table S1; see also Table S4 at https://figshare.com/articles/Supplemental_Table_4_xlsx/11873754). In the three least alkaline wells (CSW1.4, N08C, and QV1.2), most of the genes in the pathway displayed low abundance (<2 fragments per kilobase per million [FPKM]) in the metagenomic data. Metatranscriptomic data showed that the pathway was actively transcribed in all four wells that were sampled for mRNA (Fig. 2 and 5). However, one of the initial steps of the reductive acetyl-CoA pathway, the conversion of CO_2_ to formate by NADP-dependent formate dehydrogenase (FDH), was rarely expressed or altogether missing in both the metagenomes and the metatranscriptomes (Fig. 5). Seven of the 66 MAG bins also contained a nearly complete reductive acetyl-CoA pathway that was missing the genes encoding FDH (Fig. 3, bottom). The type I pathway for the formation of acetate from acetyl-CoA via acetylphosphate (present in *Methanosarcina* and *Clostridiales*) was relatively abundant across all metagenomes, while the type II pathway (which does not utilize an acetylphosphate intermediate) was sparsely represented in the metagenomic data (Fig. 2).

10.1128/mSystems.00607-19.5 TABLE S1 Key genes of interest detected across metagenomes and metatranscriptomes. Download Table S1, XLSX file, 0.1 MB. Copyright © 2020 Seyler et al. 2020 Seyler et al. This content is distributed under the terms of the Creative Commons Attribution 4.0 International license.

### Taxonomy.

Taxonomic assignments of the metagenomic contigs using PhyloPythiaS+ were consistent with previous 16S rRNA sequencing data from the CROMO wells (6) and showed that the microbial communities in QV1.1 and CSW1.1 (the two most alkaline wells) were highly similar and dominated by keystone taxa of serpentinite-hosted microbial communities, including *Comamonadaceae* and *Clostridiales* (Fig. S3). Taxonomic classification of MAG bins, including bin completeness, contamination, and strain heterogeneity, is available in Table S2; a phylogenetic tree comparing the MAGs to the three *Serpentinomonas* isolates is included in Fig. 3. Three MAGs—66, 119, and 123_1—were closely related to the three *Serpentinomonas* isolates and were classified as *Comamonadaceae*, *Hydrogenophaga* sp. (Fig. 3; Table S2).

10.1128/mSystems.00607-19.6 TABLE S2 Completeness, contamination, strain heterogeneity, and classification of MAG bins from metagenomes. Bin quality scores were assessed using CheckM; taxonomic annotations were obtained using GTDB-Tk. Download Table S2, DOCX file, 0.02 MB. Copyright © 2020 Seyler et al. 2020 Seyler et al. This content is distributed under the terms of the Creative Commons Attribution 4.0 International license.

In the metagenomes, particulate methane monooxygenase (*pmoA*) and methanol dehydrogenase (*mxaF*) genes from the methane oxidation pathway were detected on contigs classified as *Methylophilales* (*Methylophilaceae*) and *Methylococcales* (*Methylococcaceae*); two MAG bins containing a complete methane oxidation pathway were classified as *Methylococcaceae* (Fig. 3; Table S2). Genes for methylenetetrahydrofolate dehydrogenase (*folD*) and formyltetrahydrofolate deformylase (*purU*) from the THF pathway for formaldehyde oxidation were associated with *Burkholderiales* (*Comamonadaceae*), *Deinococcales* (*Trueperaceae*), *Hydrogenophilales* (*Hydrogenophilaceae*), *Methylophilales* (*Methylophilaceae*), *Rhizobiales*, and *Rhodocyclales* (*Rhodocyclaceae*). The *S*-(hydroxymethyl)glutathione dehydrogenase (*frmA*) gene from the glutathione pathway for formaldehyde oxidation was detected on contigs identified as *Burkholderiales* (*Comamonadaceae*), *Rhizobiales*, *Rhodobacterales*, and *Rhodocyclales* (*Rhodocyclaceae*). The *folD* gene is also present in the reductive acetyl-CoA pathway for acetogenesis; this gene, as well as the genes for acetyl-CoA synthase (*acsB*) and the catalytic subunit of anaerobic carbon-monoxide dehydrogenase (*cooS*), was found in *Clostridiales* (*Syntrophomodaceae*) and *Desulfuromodales*. All of the MAG bins containing the reductive acetyl-CoA pathway were classified as *Clostridiales* (Fig. 3; Table S2). The large and small subunits of RuBisCO (*rbcL* and *rbcS*) were detected on contigs classified as *Burkholderiales* (*Comamonadaceae*), *Hydrogenophilales* (*Hydrogenophilaceae*), *Rhodocyclales* (*Rhodocyclaceae*), and *Methylophilales* (*Methylophilaceae*). A complete CBB cycle was only identified in one MAG, however, classified as *Rhodocyclales* (*Rhodocyclaceae*). The ATP-citrate lyase alpha-subunit (*aclA*) gene from the reverse TCA cycle was found in *Clostridiales* (*Syntrophomodaceae*) in the metagenomes.

### Metabolomics.

**FIG 6:**
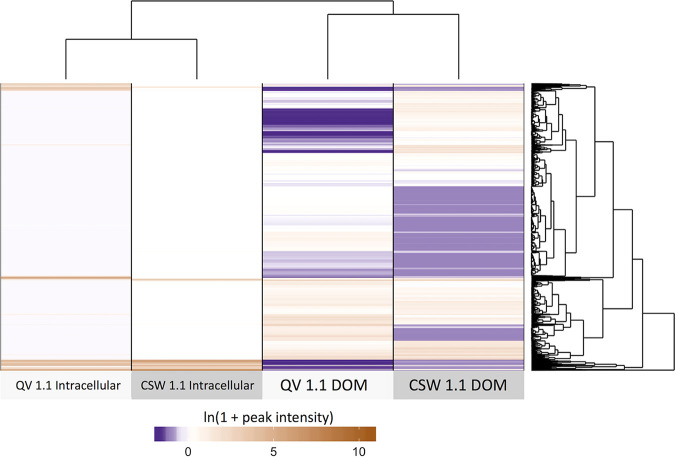
Heat map of untargeted metabolomics features across all four metabolome samples. The scale represents the ln(1 + peak intensity) [1 is added to avoid cases of ln(0)].

**FIG 7:**
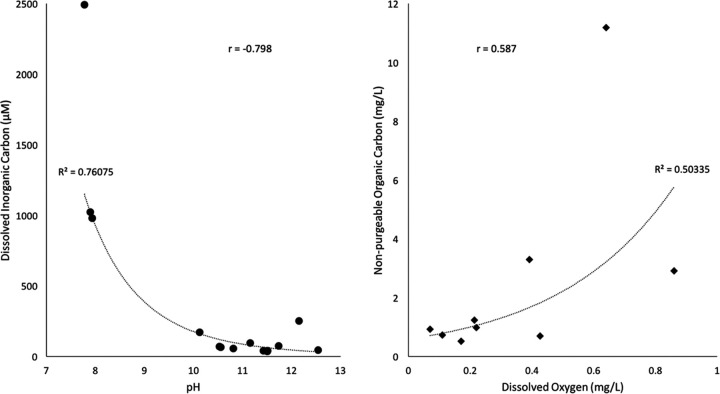
Scatterplots depicting dissolved inorganic carbon (DIC) versus pH (left) and nonpurgeable organic carbon (NPOC) versus dissolved oxygen (DO). The Pearson correlation coefficient (*r*) is indicated on each graph, as well as the *R*^2^ of the trend lines.

Metabolite extracts were obtained from both cellular biomass on filters (intracellular metabolites) and dissolved organic carbon (DOC) in the filtrate (extracellular metabolites) from the two most alkaline wells, QV1.1 and CSW1.1. The metabolomes of QV1.1 and CSW1.1 displayed many unique features across both sample types. Metabolomes had more features in common between samples of the same type than samples from the same well. In particular, the DOC pools of QV1.1 and CSW1.1 were highly distinct from one another and from intracellular extracts, and these metabolomes contained the greatest number of features unique to the sample (Fig. 6; Fig. S4). Dissolved inorganic carbon concentration in all wells was inversely proportional to pH (Pearson correlation coefficient [*r*] = −0.798), while nonpurgeable organic carbon was positively correlated to dissolved oxygen (DO) concentration (*r* = 0.587) (Fig. 7). Most of the wells in the CROMO cluster are anoxic according to the U.S. Geological Survey definition (<0.5 mg/liter), which is consistent with the hydrogen generated through the serpentinization reaction (Table S3). The correlation between bulk dissolved organic matter (DOM) and dissolved oxygen concentration could be a consequence of surficial inputs to the shallower wells, as reported in an earlier study (25). However, recent hydrogeological studies at CROMO showed that the seasonal surface-connected aquifer extends only about 7 m into the subsurface before hitting an aquitard, beneath which is a confined serpentinite aquifer (31). Additionally, subsurface tritium isotope values are below detection limits in all the wells with the exception of N08C, indicating that the waters are at least 76 years old (L. I. Putman, unpublished data). It is therefore unlikely that seasonal recharge is reaching the fluids we sampled at a rate that would be relevant for replenishing DO in the wells. All wells were below the detection limits of the colorimetric formaldehyde test kit (<0.4 ppm).

10.1128/mSystems.00607-19.7 TABLE S3 Geochemical data for all of the CROMO wells from which metagenomic/metatranscriptomic data have been obtained. Download Table S3, CSV file, 0.01 MB. Copyright © 2020 Seyler et al. 2020 Seyler et al. This content is distributed under the terms of the Creative Commons Attribution 4.0 International license.

## DISCUSSION

Previous studies of microbial communities at CROMO have used marker gene analysis, shotgun metagenomics, microcosm experiments, and geochemical measurements to elucidate pathways of biogeochemical cycling and to describe the relationship between geochemical parameters and microbial diversity (6, 25). We expanded upon these efforts by combining metagenomics and metatranscriptomics with untargeted metabolomics to verify genomic expression and metabolic activity. We also analyzed MAGs produced by combining metagenomic data across all of the wells at CROMO to assess the completeness of key pathways. Using this integrated omics approach, we found evidence of multiple carbon fixation pathways, including the Calvin-Benson-Bassham cycle, the reverse TCA cycle, acetogenesis via the reductive acetyl-CoA pathway, and carbon assimilation via methylotrophy. We also determined that these pathways were expressed by the microbial communities in the serpentinite-hosted groundwater at CROMO. These pathways were associated with several key taxa associated with serpentinite-hosted microbial communities, including *Comamonadaceae*, *Clostridiales*, *Desulfuromodales*, and *Hydrogenophilales*.

### Methane uptake and formaldehyde cycling.

The addition of methane to microcosms from CROMO has been shown to stimulate microbial growth (25). In the previous metagenomic study of 4 samples by Twing et al. (6), no *pmoA* genes were detected; however, the gene *mxaF* was identified in the CSW1.3 metagenome. Our expanded search through nine metagenomes and four metatranscriptomes succeeded in finding the genes for particulate methane monooxygenase (PMO) and methanol dehydrogenase (MXA) in the CROMO microbial communities, though at low abundance in the higher-pH, more-reducing wells (Fig. 4), suggesting that the prior perceived absence of these genes could have been the result of a smaller data set. Aerobic methanotrophy therefore appears to be more likely to occur in lower-pH, higher-E_h_ wells. Two MAG bins contain the complete methane oxidation pathway, and another two contain a partial pathway (Fig. 3). No other known methane monooxygenases or methanol dehydrogenases were identified in the metagenomes or metatranscriptomes.

The prevalence of formaldehyde and formate assimilation pathways in CROMO reinforces the importance of methylotrophy as a carbon assimilation strategy in these microbial communities. There is a wealth of metagenomic and metatranscriptomic evidence that formaldehyde oxidation to formate and formaldehyde fixation to biomass can be performed by the microbial communities in CROMO (Fig. 2). Formaldehyde is an important intermediate in methylotrophic metabolic pathways, and though it is highly toxic, methylotrophic microorganisms have developed a variety of functionally redundant modules for rapidly converting formaldehyde to less toxic compounds (32). Formaldehyde can also act as a carbon and electron shuttle between microbial subpopulations, in which it is produced by autotrophic primary producers as a by-product and then taken up by methylotrophic microbes and incorporated into biomass (33).

In methanotrophs, formate oxidation to CO_2_ is carried out by the NADP-dependent formate dehydrogenase that converts CO_2_ to formate in the Wood-Ljungdahl pathway, operating in reverse. As previously stated, the genes for this enzyme were not well represented (and in some cases missing) in the metagenomic data (Fig. 5). Formate can also be oxidized to CO_2_ by an NAD-dependent formate dehydrogenase (FDH) as part of the degradation of oxalate; however, the complete suite of genes required to produce this enzyme was likewise at low coverage or partially missing, particularly in the most alkaline, most reducing wells (Fig. S2). This contrasts with subsurface wells in the Samail Ophiolite, where FDH-encoding genes were enriched in metagenomes recovered from alkaline and hyperalkaline groundwater (29). Therefore, while there is substantial metagenomic and metatranscriptomic evidence for the production of formate from formaldehyde by the microbial communities in CROMO, DNA/RNA evidence for the further oxidation of formate to CO_2_ is lacking.

10.1128/mSystems.00607-19.2 FIG S2 Fragments per kilobase of sequences per million mapped reads of genes in the NAD-dependent pathway for formate oxidation to CO_2_. Metagenomes are represented in black; metatranscriptomes are represented in gray. Download FIG S2, PDF file, 0.1 MB. Copyright © 2020 Seyler et al. 2020 Seyler et al. This content is distributed under the terms of the Creative Commons Attribution 4.0 International license.

### Bicarbonate fixation strategies in the metagenomes and metatranscriptomes.

Despite the DIC concentrations being challengingly low for CO_2_ fixation at the high pH of the deepest wells (5, 29, 34), we found complete Calvin-Benson-Bassham (CBB; Fig. S1) and reverse tricarboxylic acid (rTCA; Fig. 2) cycles in all nine of our metagenomes and all four metatranscriptomes. Sequences corresponding to the *rbcL* gene of the RuBisCo enzyme were previously detected in metagenomes from four wells (CSW1.1, CSW1.3, QV1.1, and QV1.2) in the earlier metagenomic study by Twing et al. (6). QV1.1 displays the most abundant coverage of the rTCA pathway in both its metagenome (607.71 ± 55.83 FPKM) and metatranscriptome (446.86 ± 149.59 FPKM) (Fig. 2), even though the concentration of dissolved inorganic carbon (DIC) in QV1.1 is lower than almost any other well (11.48 μM). Conversely, the presence/expression of the CBB cycle was roughly equal throughout all the wells (Fig. S1).

The prevalence of carbon-fixing populations in these wells presents a conundrum, as above pH 11 the carbonate anion, a molecule unable to be acted upon directly by known autotrophic pathways, is the dominant species of DIC (35). *Serpentinomonas* spp. belonging to the family *Comamonadaceae* are among the most abundant taxa in the highest-pH wells in CROMO (6). RuBisCO large and small subunits (encoded by *rbcL* and *rbcS*) were detected on *Comamonadaceae* contigs in our metagenomic data, suggesting the ability to fix carbon. A previous study found that *Serpentinomonas* require the addition of calcium carbonate (even when bicarbonate is also provided) for growth in culture and form aggregates on carbonate precipitates (34). Bicarbonate-fixing microbes may create a localized microenvironment of decreased pH where carbonate may be converted to bicarbonate, or they may have bicarbonate transporters that solubilize carbonate (for examples, see reference 36). We did not detect any ATP-binding cassette transporters for bicarbonate in our metagenomic data (see Table S4 at https://figshare.com/articles/Supplemental_Table_4_xlsx/11873754). Alternatively, formate may be used as a carbon source in the CBB cycle (37) or in the rTCA cycle (38). Formate could thus be produced from formaldehyde via formaldehyde oxidation and then used in assimilatory/anabolic pathways.

Evidence for the reductive acetyl-CoA pathway was also previously detected in several wells (6). In this study, we found a nearly complete homoacetogenic reductive acetyl-CoA pathway in the metagenomic and metatranscriptomic data from all but the two least alkaline wells, except for one gene. The gene that encodes the beta subunit of formate dehydrogenase (*fdhB*), which converts CO_2_ to formate as the first step of the reductive acetyl-CoA pathway, represents <1 FPKM in every metagenome but two (N08A and N08B) (Fig. 5) and is present only in the metatranscriptomes of QV1.2 and N08B. Likewise, the genes for FDH are missing from every MAG bin that possesses the reductive acetyl-CoA pathway (Fig. 3, bottom). Yet this step of the pathway may be unnecessary for the pathway to proceed if formate is being produced by other means, either through the oxidation of formaldehyde via the glutathione-dependent or tetrahydrofolate pathway or by abiotic production via Fischer-Tropsch-like reactions. Abiotically produced formate has been implicated as a possible carbon source in serpentinizing environments such as the Lost City (14, 23, 39) and the Samail Ophiolite (29). While formate-fixing reactions are not common, as formate has a relatively low reactivity compared to that of CO_2_ or other carboxylic acids, formate can be used as the sole carbon source by some microorganisms (37). In the Samail Ophiolite, rates of assimilation or oxidation of formate were higher than rates of assimilation or dissimilation of bicarbonate or CO regardless of pH (29), suggesting that formate may be a preferred source of carbon and/or electrons. Formate concentrations in CROMO are similar to DIC concentrations or lower (Table S3; see also references 6 and 25), but the addition of formate stimulates microbial growth (25). We also detected two formate transporters in the metagenomic data: *fdhC*, which has been detected in putative formate-metabolizing bacteria in Lost City chimneys (36), was detected in two MAG bins (126 and 192, both *Clostridium*), and *focA* was detected in one MAG bin (222_8, *Actinobacter*) and across multiple metagenomes and all four metatranscriptomes (Table S1; see also Table S4 at https://figshare.com/articles/Supplemental_Table_4_xlsx/11873754).

As DIC becomes more limiting due to pH, bypassing the conversion of CO_2_ to formate may be an important strategy for carbon fixation. Provided that sufficient reductant is available (for example, from the oxidation of H_2_), assimilation of CO_2_, CO, or formate can occur via the reductive acetyl-CoA pathway (40). The assimilation of formate via the acetogenic reductive acetyl-CoA pathway avoids ATP consumption, supports energy conservation through the use of multiple electron bifurcation mechanisms, and removes the need for an external electron acceptor apart from coassimilated CO_2_ (37, 38). While the *cooS* gene for the conversion of CO_2_ to CO is present (Fig. 5), this step could likely be bypassed as well in wells where the CO concentration is sufficiently high (Table S3). Evidence for carbon monoxide uptake (28) and the reductive acetyl-CoA pathway (27) as important carbon assimilation pathways was previously found in the Tablelands, Canada, and The Cedars, CA, respectively. In the Samail Ophiolite, it appears that carbon limitation outweighs energy limitation for the autotrophic members of microbial communities at high pH (29), making carbon compounds important as both a feedstock for biosynthesis and a source of electrons.

**FIG 8:**
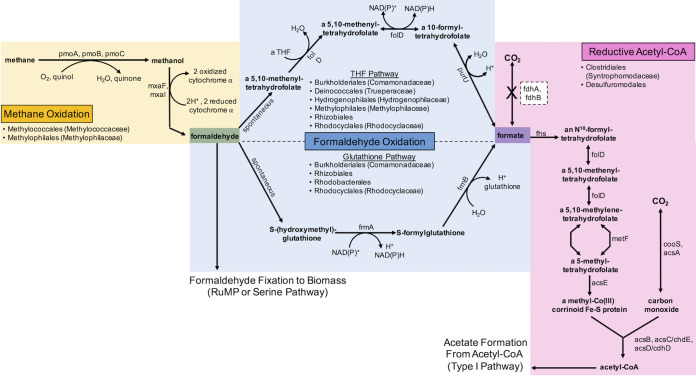
Putative pathway for carbon assimilation in the CROMO wells. Formaldehyde is either converted directly to biomass via the RuMP or serine pathway or oxidized to formate, which is then fed into the homoacetogenic reductive acetyl-CoA pathway. The pathway for methane conversion to formaldehyde is shown but appears to be rarely expressed in most of the wells.

A putative metabolic pathway for the assimilation of methane into biomass is outlined in Fig. 8. Methane oxidation to formaldehyde is carried out by the *Methylococcales* and *Methylophilales*, and then formaldehyde is either fixed to biomass via the RuMP and serine pathways or oxidized to formate via the THF (*Burkholderiales*, *Deinococcales*, *Hydrogenophilales*, *Methylophilales*, *Rhizobiales*, and *Rhodocyclales*) and glutathione-dependent (*Burkholderiales*, *Rhizobiales*, *Rhodobacterales*, and *Rhodocyclales*) pathways. Formate can then be assimilated into biomass via the reductive acetyl-CoA pathway by the *Clostridiales* or *Desulfuromodales*, as shown, or through the CBB or rTCA cycle (37, 38).

### Methanogenesis.

While methanogenic archaea have not been detected in 16S rRNA data obtained from CROMO (6), methane isotopologue data suggest a thermogenic/biogenic source (18), and metagenomic and metatranscriptomic evidence suggests the possibility of methanogenesis by multiple pathways in at least some of the wells. However, the metagenomes do not include the essential backbone of the methanogenesis pathway, including the crucial methyl coenzyme M reductase alpha subunit (*mcrA*) gene, required for making methane. The *mcrA* gene was detected in the metatranscriptomes, but other key genes for methane production were absent (Table S1; see also Table S4 at https://figshare.com/articles/Supplemental_Table_4_xlsx/11873754). Low abundances of *mcr* genes versus transcripts may be the result of methanogenesis under stress, such as hydrogen limitation (41). The presence of some methanogenesis-related genes indicates that some populations at CROMO may metabolize small organic compounds such as acetate and methanol. Whether these substrates are converted to methane by methanogens remains unclear. Biological methanogenesis appears to be occurring in other continental serpentinizing sites, such as the Voltri Massif (26) and The Cedars (19). In the Tablelands, however, no evidence of biological methane production has been detected (28), and methane production genes in metagenomes recovered from the Samail Ophiolite are likewise sparse (29). Methane cycling in ophiolite-hosted microbial communities therefore seems to vary from site to site, and further work will be required to uncover the geochemical conditions that drive its production and uptake.

### Searching the metabolome for intermediates.

Intermediates of formaldehyde oxidation and formaldehyde assimilation pathways did not match the masses and retention times of any of the features in our metabolomics data. In negative ion mode, glutathione derivatives should have appeared in the data as deprotonated or double-negatively charged molecules, but we did not detect masses that matched either of those possibilities. This may be due to the relatively low concentrations of DOM (3 to 13 mg/liter) (42), as well as bias introduced by our extraction method (43). Tetrahydrofolate compounds would likely have been more easily detected in positive ion mode; however, we ran our samples in negative mode in the hopes of capturing the broadest array of metabolites possible with no preconceived notions of what compounds would be present in the data. Metabolites may also fragment or form noncovalent interactions with other compounds upon entering the mass spectrometer, making identification of particular intermediates challenging (44).

Additionally, low cell abundances in alkaline groundwater make it difficult to obtain enough biomass during filtration to accurately depict the entire intracellular metabolome of the microbial community. An ideal target of total carbon would be 0.3 mg (45). Assuming each cell contains 30 fg of carbon (46), and with an average cell abundance of 2 × 10^5^ cells/ml (6; unpublished data), 50 liters of water would need to be filtered. Although CSW1.1 and QV1.1 have high fluid outputs compared to most of the other CROMO wells, filtration limitations such as clogging due to carbonate precipitates, keeping the fluid cold to prevent further chemical alteration of the metabolites, and prevention of contamination all precluded our ability to filter such large amounts of water. We were also unable to detect formaldehyde using colorimetric methods in any of the wells. If formaldehyde and its intermediates are cycled rapidly within the cell, it may be difficult to identify and quantify these compounds with the techniques used in this study. Interestingly, while the two wells with the highest pH (QV1.1 and CSW1.1) share highly similar microbial communities (Fig. S3) with similar metabolic capabilities, the DOC pools observed in these wells appeared significantly different in character (Fig. 6). Putative annotations of unique metabolite features in the DOC pools include dipeptides, fatty acids, vitamins, phosphorylated metabolites, and secondary metabolites. This observation underscores the need for further characterization of the available carbon in serpentinite-hosted aquifers like CROMO through metabolomics techniques.

10.1128/mSystems.00607-19.3 FIG S3 Microbial community composition of QV1.1 and CSW1.1 wells as determined by assigning taxonomy to metagenomic contigs using PhyloPython. Download FIG S3, PDF file, 0.8 MB. Copyright © 2020 Seyler et al. 2020 Seyler et al. This content is distributed under the terms of the Creative Commons Attribution 4.0 International license.

10.1128/mSystems.00607-19.4 FIG S4 Venn diagram of compounds unique to each sample and shared between samples. Observed compounds appear to be generally distinct to each sample. Filtered fluid/dissolved organic carbon samples had the greatest number of unique samples, and CSW1.1 had more unique compounds than QV1.1. Download FIG S4, PDF file, 0.1 MB. Copyright © 2020 Seyler et al. 2020 Seyler et al. This content is distributed under the terms of the Creative Commons Attribution 4.0 International license.

### Conclusion.

Through an in-depth analysis of metagenomic and metatranscriptomic data taken over several time points, we have determined that methanotrophy, the reductive acetyl-CoA pathway, the reverse TCA cycle, and the Calvin-Benson-Bassham cycle may all be important means of carbon fixation and assimilation for microbes living in the Coast Range Ophiolite groundwater. We also identified several mechanisms by which microbes in the hyperalkaline fluids of deep serpentinizing rock may overcome limiting concentrations of DIC, including the use of formate and formaldehyde as carbon sources for homoacetogenesis and the production of biomass. Further optimization of metabolomics techniques for this environment may be of use to track the fate of carbon and delineate between abiotic and biotic processes in serpentinizing systems such as CROMO.

## MATERIALS AND METHODS

### Site description.

The Coast Range Ophiolite is a 155 million- to 170 million-year-old portion of oceanic crust tectonically emplaced on the North American continental margin in northern California. Trapped Cretaceous seawater and circulating meteoric water reacts with ultramafic rock (47, 48), as evidenced by numerous calcium hydroxide rich springs throughout the formation (49). Previous studies of continental serpentinite environments have sampled similar springs in the Tablelands, Newfoundland, Canada (22, 24) and The Cedars, CA (34), providing information on the surface expression of subsurface biogeochemical processes. In order to directly access the serpentinite subsurface environment and monitor groundwater biogeochemistry with minimal contamination, a microbial observatory was established in the Coast Range Ophiolite in 2011 at the University of California (UC)-Davis McLaughlin Natural Reserve (Lower Lake, CA) (50). The Coast Range Ophiolite Microbial Observatory (CROMO) consists of two sets of wells 1.4 km apart—the Quarry Valley (QV) and Core Shed Wells (CSW)—each with an uncased main borehole (designated “1.1”) surrounded by two or four monitoring wells, respectively. In addition to the eight-well array drilled in 2011, four geographically close wells are also sampled for context: N08A, N08B, and N08C at Quarry Valley, drilled by the Homestake Mining Company, Inc., and CSWold, drilled by UC-Davis. All of the wells are cased with polyvinyl chloride (PVC), with the exception of the 1.1 wells, which are cased only to bedrock, leaving the bottom of the hole uncased. A detailed hydrogeological description of the wells is available in the work of Ortiz et al. (31).

### Extraction of DNA and RNA.

For each environmental sample from the CROMO wells, 4 liters of fluid was pumped via low-flow bladder pump (50) and immediately filtered through Sterivex 0.2-μm filter cartridges (Millipore, Billerica, MA) using a portable peristaltic pump (Masterflex, Cole Parmer, Vernon Hills, IL). Cartridges were kept on ice during filtration, immediately stored in liquid nitrogen upon completion, shipped to the home laboratory, and stored at –80°C until processing. Total genomic DNA extractions were completed as previously described by Twing et al. (6) and briefly described here. Freeze-thaw cycles and lysozyme/proteinase K treatment were performed to lyse cells, followed by purification with phenol-chloroform, precipitation using ethanol, and purification using QiaAmp (Qiagen, Hilden, Germany) columns according to the manufacturer’s instructions. A Qubit 2.0 fluorometer (Thermo Fisher, Waltham, MA) was used to quantify extracted DNA using a Qubit double-stranded DNA (dsDNA) high-sensitivity assay kit.

Extractions for RNA from CROMO wells were performed as described previously, with slight modifications (51, 52). Briefly, frozen 0.2-μm Sterivex filter cartridges were broken open, cut into four equal pieces, and divided into two screw-cap Eppendorf tubes containing phenol, 20% sodium dodecyl sulfate, 5× low-pH buffer, and 0.2 to 0.5 g of baked zirconium beads. Samples were bead-beaten for 3 min, heated in a 60°C water bath for 10 min, bead beaten again for 3 min, and centrifuged at 4°C and 18,407 × *g* to separate phases. Supernatant was transferred to a fresh Eppendorf tube and chilled. A 1× concentration of low-pH buffer was added to the remaining sample, and bead-beating was repeated. Supernatants were combined, and phenol, 1:1 phenol-chloroform, and chloroform were added in series with vortexing and centrifugation in between. Between steps, aqueous phases were transferred to clean Eppendorf tubes. The final aqueous phase was transferred to a clean Eppendorf tube with additions of ammonium acetate, isopropanol, and magnesium chloride before vortexing and incubation at –20°C overnight. Samples were centrifuged for 30 min at 4°C, washed with ethanol, and dried under vacuum before suspension in RNase-free water and storage at –80°C until analyzed.

### Metagenome and metatranscriptome analyses.

Metagenome and metatranscriptome sequencing was conducted at the Joint Genome Institute (JGI) on an Illumina HiSeq2000 instrument. A Covaris LE220 ultrasonicator was used to shear DNA samples into 270-bp fragments, and size selection was performed using solid-phase reversible immobilization (SPRI) magnetic beads (53). DNA fragments were end-repaired, A-tailed, and ligated with Illumina-compatible adapters with barcodes unique for each library. KAPA Biosystem’s next-generation sequencing library quantitative PCR (qPCR) kit and Roche LightCycler 280 reverse transcription-PCR (RT-PCR) instrument were used to quantify libraries. Ten-library pools were assembled and prepared for Illumina sequencing in one lane each. Clustered flow cells were produced using a TruSeq paired-end cluster kit (v.3) and Illumina’s cBot instrument. The Illumina HiSeq2000 instrument was utilized with a TruSeq SBS sequencing kit (v.3) and a 2 × 150 indexed run recipe to sequence the samples. The raw sequence reads were trimmed by the JGI with a minimum quality score cutoff of 10 and to remove adapters. These trimmed reads from CROMO wells were previously reported by Twing et al. (6), but additional quality filtering and a new assembly, distinct from the JGI assembly reported by Twing et al., were used for this study.

The trimmed reads from the JGI were subjected to an additional quality screen to trim 3′ adapters with cutadapt v.1.15 (54), to remove replicate sequences, and to trim sequences again with a threshold of 20 along a sliding window of 6 bases with qtrim (part of the seq-qc package [https://github.com/Brazelton-Lab/seq-qc]). All CROMO metagenomes and metatranscriptomes were pooled together for a master CROMO assembly computed with Ray Meta v.2.3.1 (55). Phylogenetic affiliation of contigs was assigned using PhyloPythiaS+ (56). Metagenome and metatranscriptome short reads were mapped to the pooled assembly using Bowtie2 v.2.2.6 (57). The Prokka pipeline (58) was used for gene prediction and functional annotation of contigs. The arguments –metagenome and –proteins were used with Prokka v.1.12 to indicate that genes should be predicted with the implementation of Prodigal v.2.6.2 (59) optimized for metagenomes as described by Twing et al. (6). Metagenome-assembled genome (MAG) bins were constructed with ABAWACA (https://github.com/CK7/abawaca) using tetranucleotide frequencies and differential abundance as measured by Bowtie2-mapped read abundances. Bin quality was computed with CheckM (60), and only high-quality MAG bins are reported here (>50% completeness and <10% contamination, as defined by Bowers et al. [61]). The completeness and contamination of some bins were improved by refinement with Binsanity (62). Taxonomic classifications of MAGs were assigned using GTDB-Tk (https://github.com/Ecogenomics/GTDBTk) (60).

Predicted protein-coding sequences were annotated by searching the Kyoto Encyclopedia of Genes and Genomes (KEGG [63, 64]) release v. 83.2 within Prokka. HTSeq v.0.6.1 (65) and were used to calculate predicted protein abundances. The abundances of predicted protein functions in all CROMO metagenomes and metatranscriptomes were normalized to predicted protein size and metagenome size. Data reported here are in units of metagenome fragments per kilobase of predicted protein sequence per million mapped reads (FPKM). Abundances of metabolic pathways were obtained by mapping KEGG protein identifiers (IDs) and their normalized counts onto the FOAM ontology (66) with MinPath (67) as implemented in HUMAnN2 v.0.6.0 (68).

The complete translated genome assemblies of *Serpentinomonas* isolates from The Cedars (*Comamonadaceae* bacterium A1, B1, and H1 [69]) were obtained as amino acid FASTA files from RefSeq (accession numbers GCF_000828895.1, GCF_000828915.1, and GCF_000696225.1). Open reading frames (ORFs) were annotated as KEGG orthologs (KOs) using BlastKOALA (70). KEGG modules were annotated within genomes using the KEGG module reconstruction tool. MAGs were translated to protein sequences using Prodigal (59) and annotated in the same manner. Modules within the three cultured representative genomes and the MAGs were then directly compared in order to compare pathways identified in available cultivars from The Cedars to pathways in the uncultured communities hosted in CROMO’s hyperalkaline groundwater. A phylogenetic tree comparing MAGs to the *Serpentinomonas* isolates was generated using SpeciesTree in KBase (71), in which relatedness is determined by alignment similarity to a selected subset of COG (Clusters of Orthologous Groups) domains and aligned using FastTree 2 (72) with 100 bootstrap replicates.

### Metabolite extraction and analysis.

Three 4-liter samples of groundwater from QV1.1 and CSW1.1 were taken in June 2016 and filtered using 0.22-μm by 47-mm polytetrafluoroethylene (PTFE) filters on a glass vacuum filtration tower. Filters were frozen in liquid nitrogen and stored at –80°C until extraction (42, 45). Approximately 30 ml of filtrate was set aside and frozen at –20°C for total organic carbon analysis on a Shimadzu total organic carbon analyzer. Formaldehyde was measured using a CHEMetrics Vacu-vials colorimetric kit (detection range, 0.4 to 8.0 ppm). The remaining filtrate was acidified to pH 2 to 3 using concentrated HCl and stored at 4°C for 5 days until further analysis by solid-phase extraction (SPE).

Frozen filters were cut into small pieces over a muffled piece of aluminum foil, using methanol-washed scissors, and then transferred into muffled amber glass vials. Two milliliters of cold extraction solvent (40:40:20 acetonitrile-methanol-0.1 M formic acid) was added to each vial, and the samples were sonicated for 10 min (73). Extracts were transferred to a centrifuge tube via pipette and centrifuged at 20,000 × *g* for 5 min at 4°C. The supernatant was then transferred to a new amber glass vial, neutralized with 6 M ammonium hydroxide, and vacuum centrifuged to dryness. Samples were dissolved in 495 μl of 95:5 water-acetonitrile with 5 μl of 5-μg/ml biotin-(ring-6,6-d_2_) added as an injection standard (42, 74).

Dissolved organics were captured from the acidified filtrate on SPE Bond Elut-PPL cartridges (Agilent Technologies) and eluted with 100% methanol into muffled amber glass vials (71, 75). SPE-PPL cartridges were rinsed with methanol immediately before use. The supernatant was passed through the cartridge using 1/8-in. by 1/4-in. PTFE tubing to pull the supernatant into the cartridge to minimize the possibility of contamination from plastic leaching, while Viton tubing was used to remove the discarded flowthrough via peristaltic pump (flow rate not exceeding 40 ml/min). The cartridges were then rinsed with at least 2 cartridge volumes of 0.01 M HCl to remove salt, and the sorbent was air dried for 5 min. Dissolved organic matter (DOM) was eluted from the cartridge with 2 ml of methanol via gravity flow into a muffled amber glass vial. The eluate was then vacuum centrifuged to dryness. Precipitated samples were dissolved in 495 μl of 95:5 water-acetonitrile, with 5 μl of 5-μg/ml biotin-(ring-6,6-d_2_).

The above-described protocol was repeated for 4 liters of Milli-Q water, and organics extracted from the filter and filtrate as described above were used as extraction blanks. A total of 495 μl of 95:5 water-acetonitrile plus 5 μl of 5-μg/ml biotin-(ring-6,6-d_2_) was also run as a blank.

Samples were analyzed using tandem liquid chromatography-mass spectrometry (LC-MS/MS) at the Michigan State University Metabolomics Core Facility. Triplicate samples from each well were separated chromatographically in a Acquity ultraperformance liquid chromatography (UPLC) BEH C_18_ column (1.7 μm; 2.1 mm by 50 mm) using a polar/nonpolar gradient made up of 10 mM TBA and 15 mM acetic acid in 97:3 water-methanol (solvent A) and 100% methanol (solvent B). The gradient was run at 99.9%/0.1% solvent A/solvent B to 1.0%/99.0% solvent A/solvent B over 9 min, held an additional 3 min at 1.0%/99.0% solvent A/solvent B, and then reversed to 99.9%/0.1% solvent A/solvent B and held another 3 min. At a rate of 400 μl/min, the sample was fed into a quadrupole time-of-flight mass spectrometer using a Waters Xevo G2-XS MS/MS in negative ion mode using a data-independent collection method (*m/z* acquisition range, 50 to 1500 Da). DOM levels were too low to allow distinction between samples and blanks from the first run, so triplicate samples were pooled and run as before. After data was converted to centroid mode, raw data files were converted from proprietary Waters RAW format into XML-based mzML format using ProteoWizard (76). Data were processed using XCMS (77) parametrized by AutoTuner (78). Isotopes and adducts of each feature were identified using CAMERA (79). This approach yielded a table of peak areas that are identified by a unique mass to charge (*m/z*) and retention time (rt) pair referred to as features. The table of features was subject to quality assurance through blank correction by removing any feature from a sample that also appeared within the blank control (described above) (80). Features were first putatively annotated as KEGG compounds using Mummichog (81). Chemical standards of putative annotations were purchased whenever available to check the retention time to increase confidence of annotation from level 1 to 2 (82). Due to the great complexity of the secondary mass spectral data and insufficient coverage of mass spectral databases for natural organic matter, MS^2^ information could not be readily used to increase confidence of annotation.

### Data availability.

CROMO metagenome and metatranscriptome sequences are publicly available in the NCBI SRA with the BioProject IDs PRJNA410019, PRJNA410020, PRJNA410022 to PRJNA410033, PRJNA410035 to PRJNA410037, PRJNA410054, PRJNA410057, PRJNA410286, PRJNA410403, PRJNA410404, PRJNA410553 to PRJNA410555, and PRJNA410557. Raw spectrometry data are available in the MetaboLights database under study identifier MTBLS1260.
