# Supplementary figures and images for: Carbon Assimilation Strategies in Ultrabasic Groundwater: Clues from the Integrated Study of a Serpentinization-Influenced Aquifer
Source: mSystems. 2020 Mar 10;5(2):e00607-19. doi: 10.1128/mSystems.00607-19 (PMC7065513; doi:10.1128/mSystems.00607-19)

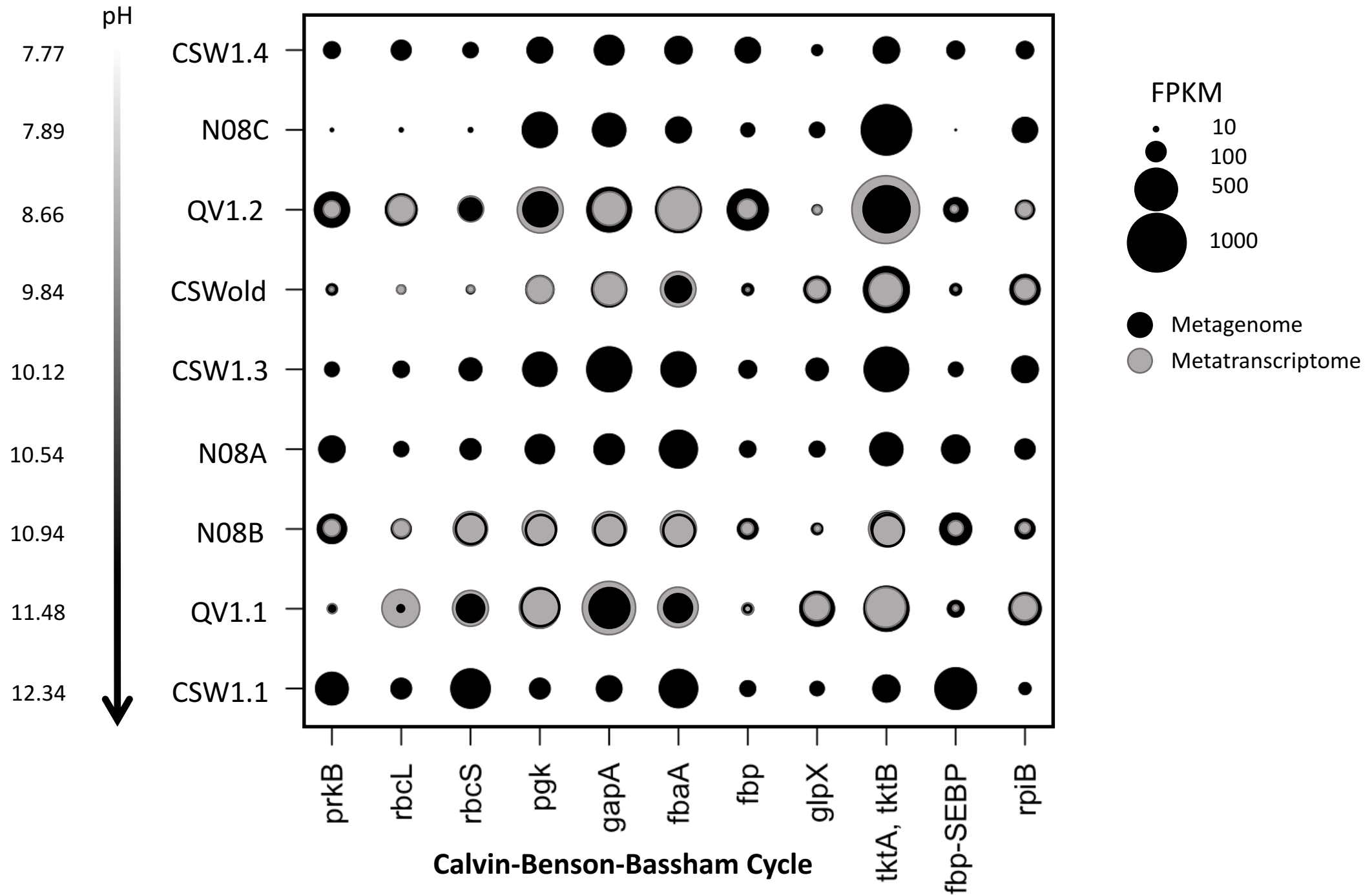

Supplement: FIG S1 [file mSystems.00607-19-sf001.pdf]

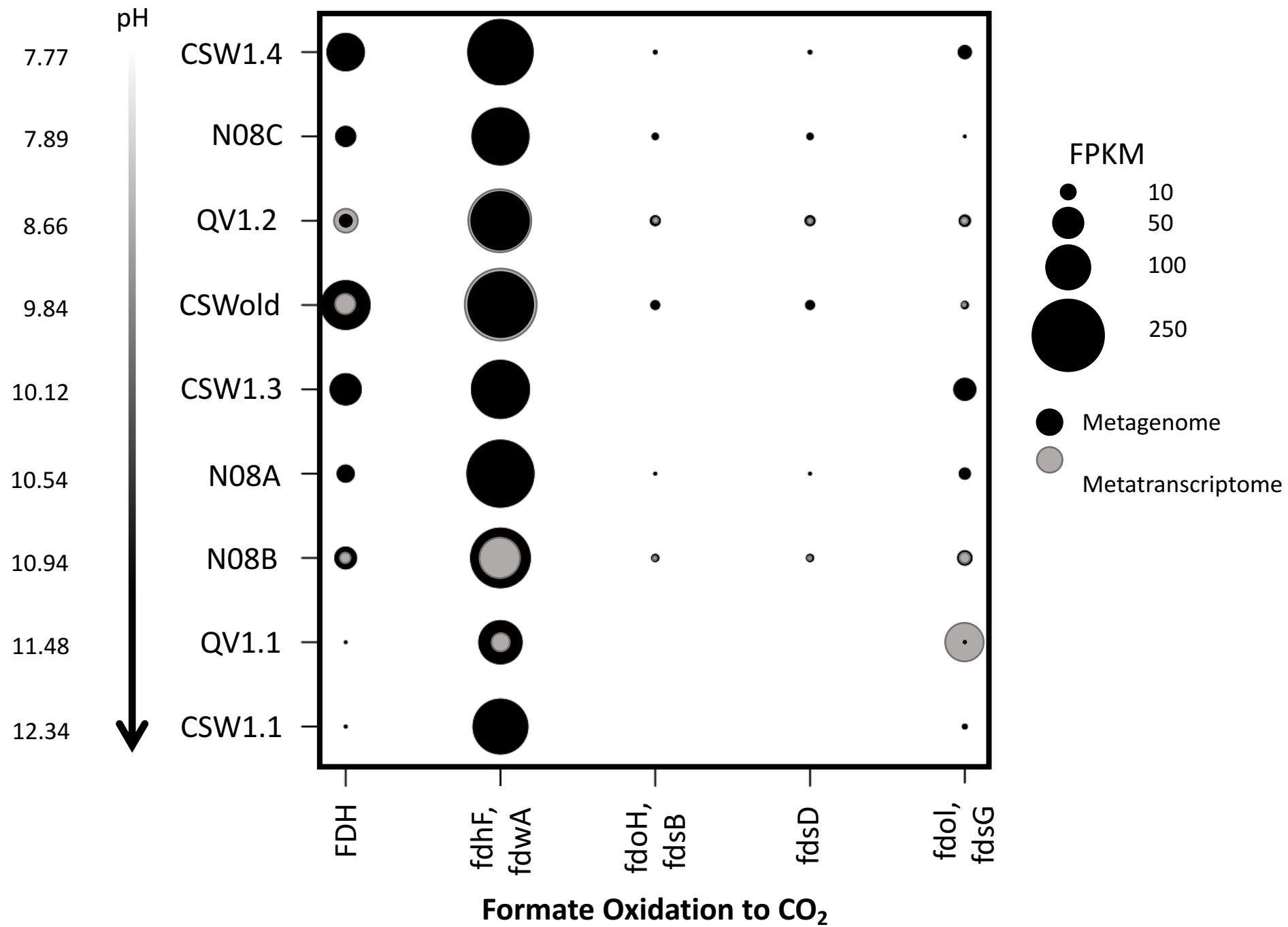

Supplement: FIG S2 [file mSystems.00607-19-sf002.pdf]

## Metagenomes

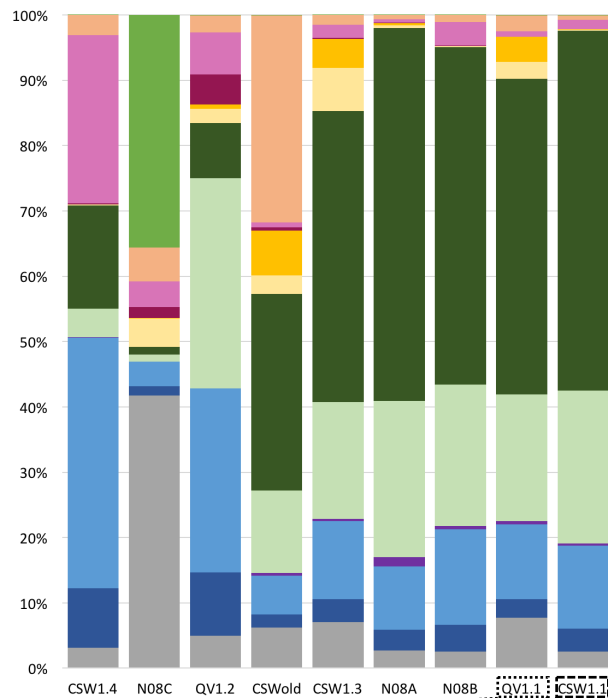

## Metatranscriptomes

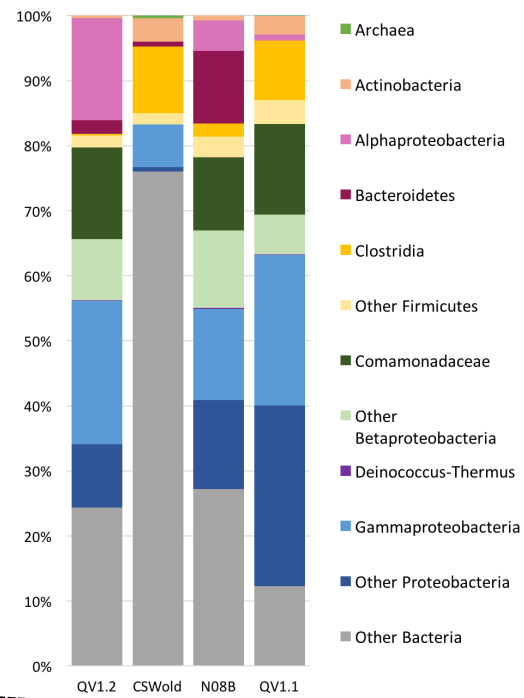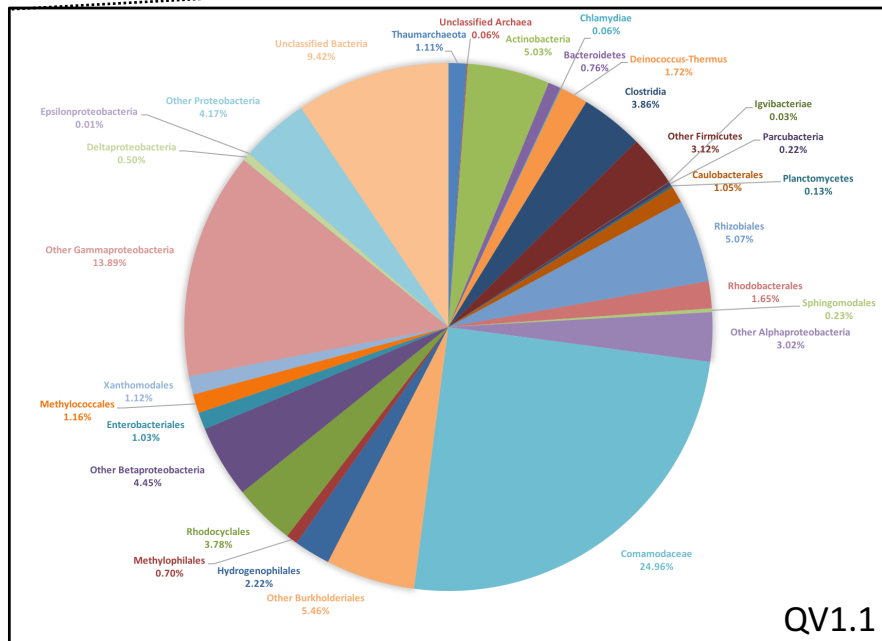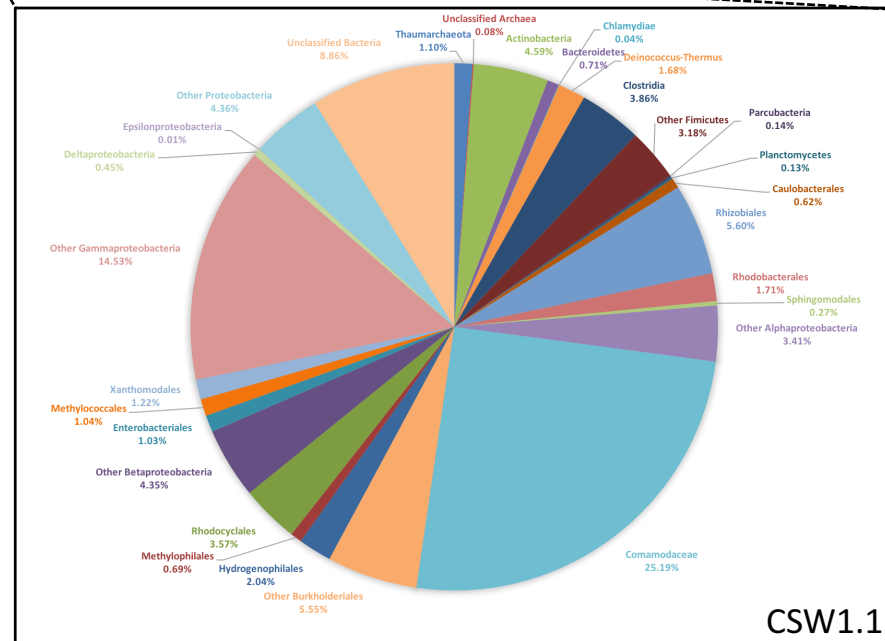

Supplement: FIG S3 [file mSystems.00607-19-sf003.pdf]

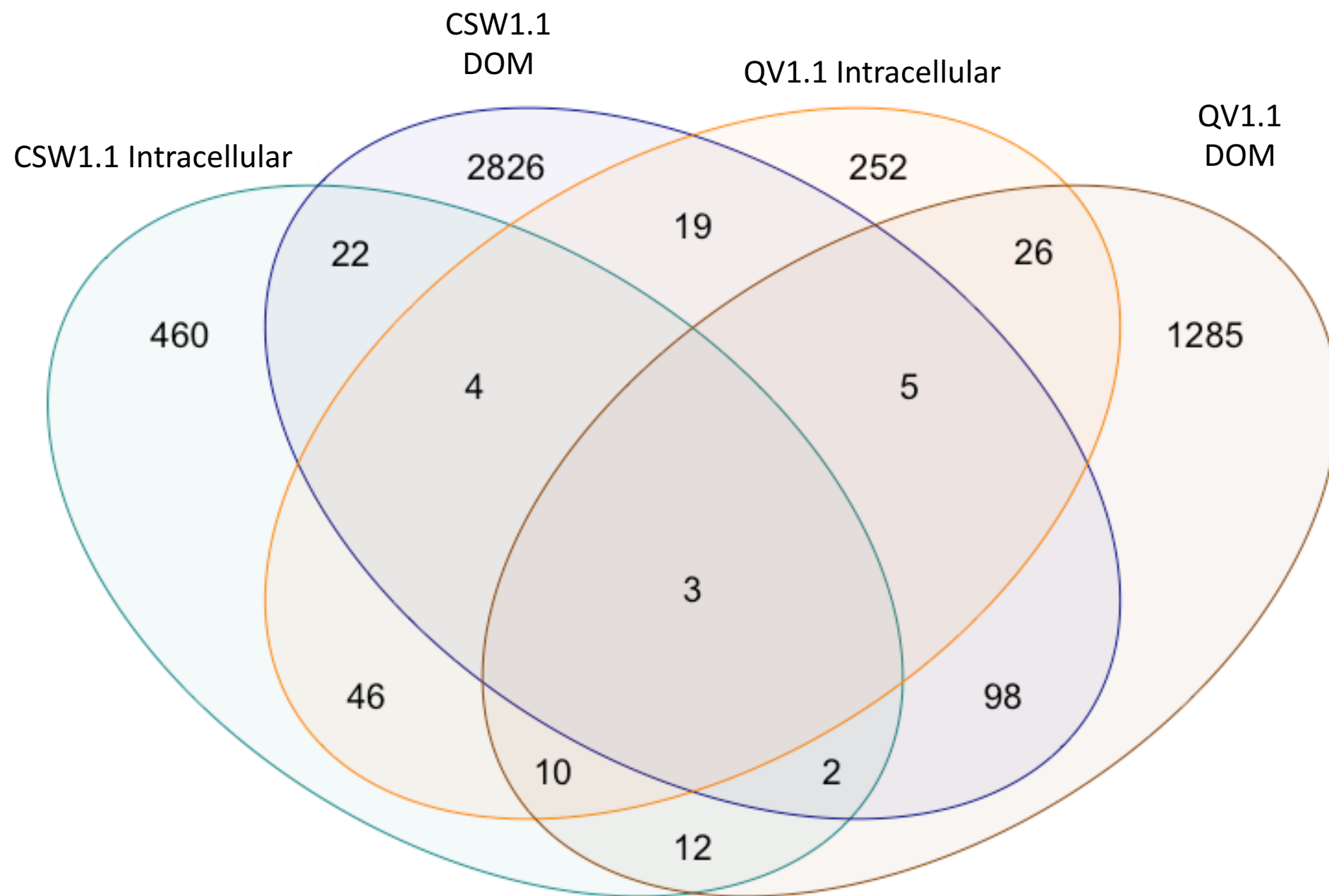

Supplement: FIG S4 [file mSystems.00607-19-sf004.pdf]
