# Supplementary material for: Carbon Assimilation Strategies in Ultrabasic Groundwater: Clues from the Integrated Study of a Serpentinization-Influenced Aquifer
Source: mSystems. 2020 Mar 10;5(2):e00607-19. doi: 10.1128/mSystems.00607-19 (PMC7065513; doi:10.1128/mSystems.00607-19)
Supplement: TABLE S2 [file mSystems.00607-19-st002.docx]

| MAG | Complete-ness | | | Conta-mina-tion | | | Strain hetero-geneity | | | Domain | | | Phylum | | | Class | | | Order | | | | | Family | | | | Genus | | | | | | | Species | | | | | | | | | | | | | | | | | | | |  |  |  |  |  |  |  |  |  |  |  |  |  |  |  |  |  |  |  |  |  |  |  |  |  |  |  |
| --- | --- | --- | --- | --- | --- | --- | --- | --- | --- | --- | --- | --- | --- | --- | --- | --- | --- | --- | --- | --- | --- | --- | --- | --- | --- | --- | --- | --- | --- | --- | --- | --- | --- | --- | --- | --- | --- | --- | --- | --- | --- | --- | --- | --- | --- | --- | --- | --- | --- | --- | --- | --- | --- | --- | --- | --- | --- | --- | --- | --- | --- | --- | --- | --- | --- | --- | --- | --- | --- | --- | --- | --- | --- | --- | --- | --- | --- | --- | --- | --- | --- |
| 83_0_0 | 73.25 | | | 3.56 | | | 20 | | | Archaea | | | Thaumarchaeota | | | incertae sedis | | | Nitrosopumilales | | | | | Nitrosopumilaceae | | | | Nitrosoarchaeum | | | | | | | Candidatus  Nitrosoarchaeum koreensis | | | | | | | | | | | | | | | | |  |  |  |  |  |  |  |  |  |  |  |  |  |  |  |  |  |  |  |  |  |  |  |  |  |  |  |  |  |  |
| 83_3_0 | 91.75 | | | 4.85 | | | 100 | | | Archaea | | | Thaumarchaeota | | | incertae sedis | | | Nitrosopumilales | | | | | Nitrosopumilaceae | | | | Nitrosoarchaeum | | | | | | | | | | | Candidatus  Nitrosoarchaeum koreensis | | | | | | | | | | | | | |  |  |  |  |  |  |  |  |  |  |  |  |  |  |  |  |  |  |  |  |  |  |  |  |  |  |  |  |  |
| 109_2_0 | 75.96 | | | 0.85 | | | 100 | | | Bacteria | | | Actinobacteria | | | Actinobacteria | | | | | | | | |  | | | | |  | | | | | | | |  | | | | | | | | | | | | | | | | | | | | | | |  |  |  |  |  |  |  |  |  |  |  |  |  |  |  |  |  |  |  |  |  |
| 222_8 | 97.01 | | | 2.56 | | | 0 | | | Bacteria | | | Actinobacteria | | | Actinobacteria | | | | | | | | |  | | | | |  | | | | | | | |  | | | | | | | | | | | | | | | | | | | | | | |  |  |  |  |  |  |  |  |  |  |  |  |  |  |  |  |  |  |  |  |  |
| 152 | 94.17 | | | 1.11 | | | 0 | | | Bacteria | | | Actinobacteria | | | Actinobacteria | | | | | | | | |  | | | | |  | | | | | | | |  | | | | | | | | | | | | | | | | | | | | | | |  |  |  |  |  |  |  |  |  |  |  |  |  |  |  |  |  |  |  |  |  |
| 58 | 98.5 | | | 3.33 | | | 47.37 | | | Bacteria | | | Bacteroidetes | | | Sphingobacteria | | | | Sphingobacteriales | | | | | Cyclobacteriaceae | | | | Algoriphagus | | | | | | | Algoriphagus  machipongonensis | | | | | | | | | | | | | | |  |  |  |  |  |  |  |  |  |  |  |  |  |  |  |  |  |  |  |  |  |  |  |  |  |  |  |  |  |  |  |
| 11 | 94.62 | | | 5.41 | | | 29.41 | | | Bacteria | | | Bacteroidetes | | | Bacteroidia | | | | Bacteroidales | | | | | | | | |  | | | | | | |  | | | | | | | | | | | | | | | | | |  |  |  |  |  |  |  |  |  |  |  |  |  |  |  |  |  |  |  |  |  |  |  |  |  |  |  |  |
| 82 | 94.83 | | | 4.15 | | | 69.23 | | | Bacteria | | | Bacteroidetes | | | Chitinophagia | | | | Chitinophagales | | | | | Chitinophagaceae | | | | | | | | | | | | | | |  | | | | | | | | | | | | | | | | | | | |  |  |  |  |  |  |  |  |  |  |  |  |  |  |  |  |  |  |  |  |  |  |
| 49 | 78.79 | | | 7.76 | | | 9.09 | | | Bacteria | | | Bacteroidetes | | | Chitinophagia | | | | Chitinophagales | | | | | Chitinophagaceae | | | | | | | | | | | | | | |  | | | | | | | | | | | | | | | | | | | |  |  |  |  |  |  |  |  |  |  |  |  |  |  |  |  |  |  |  |  |  |  |
| 74 | 97.7 | | | 6.12 | | | 54.17 | | | Bacteria | | | Bacteroidetes | | | Flavobacteriia | | | | Flavobacteriales | | | | | | | | |  | | | | | | |  | | | | | | | | | | | | | | | | | | | |  |  |  |  |  |  |  |  |  |  |  |  |  |  |  |  |  |  |  |  |  |  |  |  |  |  |
| 85_0 | 96.21 | | | 4.08 | | | 0 | | | Bacteria | | | Bacteroidetes | | | | | | | |  | | | |  | | | | | |  | | | | | | | | | | | | | | | | | |  | | | | | | | | | | | | | | | | | | | | | | | | | | | |  |  |  |  |  |
| 64 | 89.73 | | | 4.12 | | | 38.46 | | | Bacteria | | | Bacteroidetes | | | | | | | |  | | | |  | | | | | |  | | | | | | | | | | | | | | | | | |  | | | | | | | | | | | | | | | | | | | | | | | | | | | |  |  |  |  |  |
| 120 | 85.67 | | | 0.26 | | | 0 | | | Bacteria | | | Bacteroidetes | | | | | | | |  | | | |  | | | | | |  | | | | | | | | | | | | | | | | | |  | | | | | | | | | | | | | | | | | | | | | | | | | | | |  |  |  |  |  |
| 117 | 97.31 | | | 5.38 | | | 14.29 | | | Bacteria | | | Bacteroidetes | | | | | | | |  | | | |  | | | | | |  | | | | | | | | | | | | | | | | | |  | | | | | | | | | | | | | | | | | | | | | | | | | | | |  |  |  |  |  |
| 87_3_0 | 84.08 | | | 2.7 | | | 20 | | | Bacteria | | | Chlamydiae | | | Chlamydiae | | | | | Chlamydiales | | | | Simkaniaceae | | | | | | Simkania | | | | | Simkania negevensis | | | | | | | | | | | | | | | | | | | | |  |  |  |  |  |  |  |  |  |  |  |  |  |  |  |  |  |  |  |  |  |  |  |  |  |
| 100 | 98.88 | | | 8.45 | | | 0 | | | Bacteria | | | Chlorobi | | | Ignavibacteria | | | | | Ignavibacteriales | | | | Ignavibacteriaceae | | | | | | Ignavibacterium | | | | | Ignavibacterium album | | | | | | | | | | | | | | | | | | | | |  |  |  |  |  |  |  |  |  |  |  |  |  |  |  |  |  |  |  |  |  |  |  |  |  |
| 200 | 84.69 | | | 1.12 | | | 0 | | | Bacteria | | | Chlorobi | | | Ignavibacteria | | | | | Ignavibacteriales | | | | Melioribacteraceae | | | | | | Melioribacter | | | | | Melioribacter roseus | | | | | | | | | | | | | | | | | | | | |  |  |  |  |  |  |  |  |  |  |  |  |  |  |  |  |  |  |  |  |  |  |  |  |  |
| 84 | 71.17 | | | 1.09 | | | 0 | | | Bacteria | | | Chlorobi | | | Ignavibacteria | | | | | Ignavibacteriales | | | | | | | | | |  | | | | |  | | | | | | | | | | | | | | | | | | | | | | | | | | | |  |  |  |  |  |  |  |  |  |  |  |  |  |  |  |  |  |  |
| 191 | 88.56 | | | 5.59 | | | 50 | | | Bacteria | | | Deinococcus-Thermus | | | Deinococci | | | | | Deinococcales | | | | Trueperaceae | | | | | | Truepera | | | | | | | | | | | | | | Truepera radiovictrix | | | | | | | | | | | | | |  |  |  |  |  |  |  |  |  |  |  |  |  |  |  |  |  |  |  |  |  |  |  |
| 207 | 59.14 | | | 2.33 | | | 90 | | | Bacteria | | | Deinococcus-Thermus | | | Deinococci | | | | | Deinococcales | | | | Trueperaceae | | | | | | Truepera | | | | | | | | | | | | | | Truepera radiovictrix | | | | | | | | | | | | | |  |  |  |  |  |  |  |  |  |  |  |  |  |  |  |  |  |  |  |  |  |  |  |
| 126 | 51.67 | | | 1.4 | | | 100 | | | Bacteria | | | Firmicutes | | | Clostridia | | | | | Clostridiales | | | | Clostridiaceae | | | | | | Alkaliphilus | | | | | | | | | | | | | | | | | | | | | | | | | | |  |  |  |  |  |  |  |  |  |  |  |  |  |  |  |  |  |  |  |  |  |  |  |  |
| 128 | 94.07 | | | 3.11 | | | 68.75 | | | Bacteria | | | Firmicutes | | | Clostridia | | | | | Clostridiales | | | | Clostridiaceae | | | | | | Clostridium | | | | | | | | | | | | | | | | | | | | | | | | | | | | | | | | | |  |  |  |  |  |  |  |  |  |  |  |  |  |  |  |  |  |
| 61 | 93.27 | | | 6.73 | | | 75 | | | Bacteria | | | Firmicutes | | | Clostridia | | | | | Clostridiales | | | | Clostridiales Family XVII. Incertae Sedis | | | | | | Thermaerobacter | | | | | | | | | | | | | | | | | | | | | | | | | | | | | | | | | |  |  |  |  |  |  |  |  |  |  |  |  |  |  |  |  |  |
| 245 | 94.23 | | | 0.24 | | | 0 | | | Bacteria | | | Firmicutes | | | Clostridia | | | | | Clostridiales | | | | Peptococcaceae | | | | | | | | | | | | | | | | | | | | | | |  | | | | | | | | | | | | | | | | | | | | | |  |  |  |  |  |  |  |  |  |  |  |  |
| 236 | 80.27 | | | 0.32 | | | 0 | | | Bacteria | | | Firmicutes | | | Clostridia | | | | | Clostridiales | | | | Peptococcaceae | | | | | | | | | | | | | | | | | | | | | | |  | | | | | | | | | | | | | | | | | | | | | |  |  |  |  |  |  |  |  |  |  |  |  |
| 244 | 54 | | | 3.25 | | | 100 | | | Bacteria | | | Firmicutes | | | Clostridia | | | | | Clostridiales | | | | Syntrophomonadaceae | | | | | | Dethiobacter | | | | | | | | | | | | | | | | | | | Dethiobacter alkaliphilus | | | | | | | | | | | | | | | | | | | | | |  |  |  |  |  |  |  |  |  |  |
| 105 | 94.35 | | | 4.31 | | | 20 | | | Bacteria | | | Firmicutes | | | Clostridia | | | | | Clostridiales | | | | Syntrophomonadaceae | | | | | | Dethiobacter | | | | | | | | | | | | | | | | | | | Dethiobacter alkaliphilus | | | | | | | | | | | | | | | | | | | | | |  |  |  |  |  |  |  |  |  |  |
| 148 | 81.81 | | | 1.55 | | | 40 | | | Bacteria | | | Firmicutes | | | Clostridia | | | | | Clostridiales | | | | Syntrophomonadaceae | | | | | | Dethiobacter | | | | | | | | | | | | | | | | | | | Dethiobacter alkaliphilus | | | | | | | | | | | | | | | | | | | | | |  |  |  |  |  |  |  |  |  |  |
| 151 | 89.75 | | | 3.95 | | | 50 | | | Bacteria | | | Firmicutes | | | Clostridia | | | | | Clostridiales | | | | Syntrophomonadaceae | | | | | | Dethiobacter | | | | | | | | | | | | | | | | | | | Dethiobacter alkaliphilus | | | | | | | | | | | | | | | | | | | | | |  |  |  |  |  |  |  |  |  |  |
| 243 | 80.51 | | | 3.39 | | | 0 | | | Bacteria | | | Firmicutes | | | Clostridia | | | | | Clostridiales | | | | Syntrophomonadaceae | | | | | | Dethiobacter | | | | | | | | | | | | | | | | | | | Dethiobacter alkaliphilus | | | | | | | | | | | | | | | | | | | | | |  |  |  |  |  |  |  |  |  |  |
| 165 | 96.41 | | | 1.28 | | | 33.33 | | | Bacteria | | | Firmicutes | | | Clostridia | | | | | Clostridiales | | | | Syntrophomonadaceae | | | | | | | | | | | | | | | | | | | | | | | | | | | | | | | | | | | | | | | | |  |  |  |  |  |  |  |  |  |  |  |  |  |  |  |  |
| 104 | 95.76 | | | 1.41 | | | 0 | | | Bacteria | | | Firmicutes | | | Clostridia | | | | | Clostridiales | | | | | | | | | |  | | | | | | | | | | | | | | | |  | | | | | | | | | | | | | | | | | | | | | | | | | | | | |  |  |  |  |  |  |
| 182 | 94.83 | | | 3.33 | | | 0 | | | Bacteria | | | Firmicutes | | | Clostridia | | | | | Clostridiales | | | | | | | | | |  | | | | | | | | | | | | | | | |  | | | | | | | | | | | | | | | | | | | | | | | | | | | | |  |  |  |  |  |  |
| 192 | 78.72 | | | 2.07 | | | 50 | | | Bacteria | | | Firmicutes | | | Clostridia | | | | |  | | | |  | | | | | |  | | | | | | | | | | | | | | | | | | |  | | | | | | | | | | | | | | | | | | | | | | | | | | | |  |  |  |  |
| 107 | 79.91 | | | 3.49 | | | 0 | | | Bacteria | | | Firmicutes | | | Clostridia | | | | |  | | | |  | | | | | |  | | | | | | | | | | | | | | | | | | |  | | | | | | | | | | | | | | | | | | | | | | | | | | | |  |  |  |  |
| 142 | 89.74 | | | 0.94 | | | 0 | | | Bacteria | | | Firmicutes | | | Erysipelotrichia | | | | | Erysipelotrichales | | | | Erysipelotrichaceae | | | | | | Erysipelothrix | | | | | | | | | | | | | | | | | | | | | | | | | | | | | | | | | | | | | | | | | | | | | | | | |  |  |
| 134 | 87.57 | | | 8.49 | | | 81.82 | | | Bacteria | | | Firmicutes | | | Erysipelotrichia | | | | | Erysipelotrichales | | | | Erysipelotrichaceae | | | | | | Erysipelothrix | | | | | | | | | | | | | | | | | | | | | | | | | | | | | | | | | | | | | | | | | | | | | | | | |  |  |
| 155 | 100 | | | 5.66 | | | 37.5 | | | Bacteria | | | Firmicutes | | | Erysipelotrichia | | | | | Erysipelotrichales | | | | Erysipelotrichaceae | | | | | | Erysipelothrix | | | | | | | | | | | | | | | | | | | | | | | | | | | | | | | | | | | | | | | | | | | | | | | | | | |
| 147 | 95.19 | | | 0.74 | | | 75 | | | Bacteria | | | Firmicutes | | | Negativicutes | | | | | Selenomonadales | | | | | | | | | |  | | | | | | | | | | | | | | | |  | | | | | | | | | | | | | | | | | | | | | | | | | | | | |  |  |  |  |  |  |
| 102 | 91.11 | | | 1.72 | | | 0 | | | Bacteria | | | Latescibacteria | | | | | | | |  | | | |  | | | | | |  | | | | | | | | | | | | | | | | | |  | | | | | | | | | | | | | | | | | | | | | | | | | | | |  |  |  |  |  |
| 145 | 92.78 | | | 3.7 | | | 83.33 | | | Bacteria | | | Proteobacteria | | | Alphaproteobacteria | | | | | Rhizobiales | | | | Bradyrhizobiaceae | | | | | | Afipia | | | | | | | Afipia broomeae | | | | | | | | | | | | | | | | | | | | | | | | | | | | | |  |  |  |  |  |  |  |  |  |  |  |  |  |  |
| 2 | 88.68 | | | 0.95 | | | 0 | | | Bacteria | | | Proteobacteria | | | Alphaproteobacteria | | | | | Rhizobiales | | | | | | | | | |  | | | | | | |  | | | | | | | | | | | | | | | | | | | | | | | | | | | | | |  |  |  |  |  |  |  |  |  |  |  |  |  |  |
| 118 | 52.2 | | | 1.18 | | | 0 | | | Bacteria | | | Proteobacteria | | | Alphaproteobacteria | | | | | Rhodobacterales | | | | Rhodobacteraceae | | | | | | Roseovarius | | | | | | | Roseovarius  sp. TM1035 | | | | | | | | | | | | | | | | | | | | | | | | | | | | | |  |  |  |  |  |  |  |  |  |  |  |  |  |  |
| 187 | 94.42 | | | 0.53 | | | 25 | | | Bacteria | | | Proteobacteria | | | Alphaproteobacteria | | | | | Rhodobacterales | | | | Rhodobacteraceae | | | | | | | | | | | | | | | | | | | | |  | | | | | | | | | | | | | | | | | | | | | | | | | | | |  |  |  |  |  |  |  |  |
| 87_2_1 | 69.96 | | | 6.9 | | | 0 | | | Bacteria | | | Proteobacteria | | | Alphaproteobacteria | | | | | Rickettsiales | | | | Anaplasmataceae | | | | | | | | | | | | | | | | | | | | |  | | | | | | | | | | | | | | | | | | | | | | | | | | | |  |  |  |  |  |  |  |  |
| 188 | 88.47 | | | 7.97 | | | 65.22 | | | Bacteria | | | Proteobacteria | | | Alphaproteobacteria | | | | | Sphingomonadales | | | | Sphingomonadaceae | | | | | | Sandarakinorhabdus | | | | | | | | Sandarakinorhabdus  sp. AAP62 | | | | | | | | | | | | | | | | | | | | | | | | | | | | | |  |  |  |  |  |  |  |  |  |  |  |  |  |
| 53_5 | 51.72 | | | 0 | | | 0 | | | Bacteria | | | Proteobacteria | | | Alphaproteobacteria | | | | | Sphingomonadales | | | | Sphingomonadaceae | | | | | | | | | | | | | | | | | | | | | | | | | | | | | | | | | | | | | | | | | | | | | | | | | |  |  |  |  |  |  |  |
| 53_1 | 89.43 | | | 0.39 | | | 50 | | | Bacteria | | | Proteobacteria | | | Alphaproteobacteria | | | | | Sphingomonadales | | | | | | | | | |  | | | | | | | | | | | | | | | |  | | | | | | | | | | | | | | | | | | | | | | | | | | | | |  |  |  |  |  |  |
| 67 | 97.33 | | | 0 | | | 0 | | | Bacteria | | | Proteobacteria | | | Alphaproteobacteria | | | | | | | | |  | | | | | | | | |  | | | | | | | |  | | | | | | | | | | | | | | | | | | | | | | | | | | | | |  |  |  |  |  |  |  |  |  |  |  |
| 66 | | 66.03 | | | 3.31 | | | 100 | | | Bacteria | | | Proteobacteria | | | Betaproteobacteria | | | | | Burkholderiales | | | | Comamonadaceae | | | | | | Hydrogenophaga | | | | Hydrogenophaga sp. PBC | | | | | | | | | | | | | | | | | | | | | | | | | | | | |  |  |  |  |  |  |  |  |  |  |  |  |  |  |  |  |  |
| 119 | | 59.64 | | | 0 | | | 0 | | | Bacteria | | | Proteobacteria | | | Betaproteobacteria | | | | | Burkholderiales | | | | Comamonadaceae | | | | | | Hydrogenophaga | | | | Hydrogenophaga sp. PBC | | | | | | | | | | | | | | | | | | | | | | | | | | | | |  |  |  |  |  |  |  |  |  |  |  |  |  |  |  |  |  |
| 123_1 | | 54.83 | | | 0.23 | | | 100 | | | Bacteria | | | Proteobacteria | | | Betaproteobacteria | | | | | Burkholderiales | | | | Comamonadaceae | | | | | | Hydrogenophaga | | | | Hydrogenophaga sp. PBC | | | | | | | | | | | | | | | | | | | | | | | | | | | | |  |  |  |  |  |  |  |  |  |  |  |  |  |  |  |  |  |
| 6 | | 93.81 | | | 6.35 | | | 5 | | | Bacteria | | | Proteobacteria | | | Betaproteobacteria | | | | | Nitrosomonadales | | | | Gallionellaceae | | | | | | Sulfuricella | | | | Sulfuricella denitrificans | | | | | | | | | | | | | | | | | | | | | | | | | | | | |  |  |  |  |  |  |  |  |  |  |  |  |  |  |  |  |  |
| 22_9 | | 57.52 | | | 0 | | | 0 | | | Bacteria | | | Proteobacteria | | | Betaproteobacteria | | | | | Nitrosomonadales | | | | Sterolibacteriaceae | | | | | | Sulfuritalea | | | | Sulfuritalea  hydrogenivorans | | | | | | | | | | | | | | | | | | | | | | | | | | | | |  |  |  |  |  |  |  |  |  |  |  |  |  |  |  |  |  |
| 203 | | 77.21 | | | 0.36 | | | 66.67 | | | Bacteria | | | Proteobacteria | | | Betaproteobacteria | | | | | Nitrosomonadales | | | | Sterolibacteriaceae | | | | | | Sulfuritalea | | | | Sulfuritalea  hydrogenivorans | | | | | | | | | | | | | | | | | | | | | | | | | | | | |  |  |  |  |  |  |  |  |  |  |  |  |  |  |  |  |  |
| 93 | | 95.73 | | | 6.56 | | | 10 | | | Bacteria | | | Proteobacteria | | | Betaproteobacteria | | | | | Rhodocyclales | | | | Rhodocyclaceae | | | | | | Dechloromonas | | | | Dechloromonas aromatica | | | | | | | | | | | | | | | | | | | | | | | | | | | | |  |  |  |  |  |  |  |  |  |  |  |  |  |  |  |  |  |
| 116 | | 96.34 | | | 5.19 | | | 18.18 | | | Bacteria | | | Proteobacteria | | | Deltaproteobacteria | | | | | Desulfobacterales | | | | Desulfobulbaceae | | | | | | | | | |  | | | | | | | | | | | | | | | | | | | | | | | | | | |  |  |  |  |  |  |  |  |  |  |  |  |  |  |  |  |  |  |  |
| 212_2_0 | | | 76.16 | | | 1.29 | | | 0 | | | Bacteria | | | Proteobacteria | | | Deltaproteobacteria | | | | | Syntrophobacterales | | | | Syntrophaceae | | | | | | | | | | | | | | | | |  | | | | | | | | | | | | | | | | | | | | | | | | | | | | |  |  |  |  |  |  |  |  |  |
| 46_3 | | | 90.84 | | | 3.23 | | | 0 | | | Bacteria | | | Proteobacteria | | | Deltaproteobacteria | | | | | | | | |  | | | | | |  | | | | | | | |  | | | | | | | | | | | | | | | | | | | | | | | | | | | | | | | |  |  |  |  |  |  |  |  |  |
| 10 | | | 99.59 | | | 2.78 | | | 0 | | | Bacteria | | | Proteobacteria | | | Epsilonproteobacteria | | | | | Campylobacterales | | | | Helicobacteraceae | | | | | | Sulfurimonas | | | | Sulfurimonas denitrificans | | | | | | | | | | | | | | | | | | | | | | | | | | | | | | | | | | | | | | | | | |  |  |  |
| 115_2 | | | 98.56 | | | 3.12 | | | 0 | | | Bacteria | | | Proteobacteria | | | Gammaproteobacteria | | | | | Methylococcales | | | | Methylococcaceae | | | | | | Methylomicrobium | | | | | | | | | | Methylomicrobium  buryatense | | | | | | | | | | | | | | | | | | | | | | | | | | | | | | | | | | | | | | |
| 70 | | | 74.66 | | | 2.24 | | | 0 | | | Bacteria | | | Proteobacteria | | | Gammaproteobacteria | | | | | Methylococcales | | | | Methylococcaceae | | | | | | Methylomonas | | | | Methylomonas methanica | | | | | | | | | | | | | | | | | | | | | | | | | | | | | | | | | | | | | | | | | | | |  |
| 173 | | | 87.07 | | | 6.58 | | | 0 | | | Bacteria | | | Proteobacteria | | | Gammaproteobacteria | | | | | Methylococcales | | | | Methylococcaceae | | | | | | Methylomonas | | | | | | | | | | | | | | | | | | | | | | | | | | | | |  |  |  |  |  |  |  |  |  |  |  |  |  |  |  |  |  |  |  |  |
| 196 | | | 89.46 | | | 3.62 | | | 31.58 | | | Bacteria | | | Proteobacteria | | | Gammaproteobacteria | | | | | Xanthomonadales | | | | Xanthomonadaceae | | | | | | | | | |  | | | | | | | | | | | | | | | | | | | | | | | | | | | | | |  |  |  |  |  |  |  |  |  |  |  |  |  |  |  |
| 221_12 | | | 53.45 | | | 0 | | | 0 | | | Bacteria | | | Proteobacteria | | | Gammaproteobacteria | | | | | Xanthomonadales | | | | Xanthomonadaceae | | | | | | | | | |  | | | | | | | | | | | | | | | | | | | | | | | | | | | | | |  |  |  |  |  |  |  |  |  |  |  |  |  |  |  |
| 91 | | | 64.08 | | | 5.39 | | | 0 | | | Eukaryota | | | Loukozoa | | | Malawimonadea | | | | | Malawimonadida | | | | Malawimonadidae | | | | | | Malawimonas | | | | | | | | Malawimonas jakobiformis | | | | | | | | | | | | | | | | | | | | | | | | | | | | | | | | | | | | | | | |  |
